# Supplementary material for: Splice-Junction-Based Mapping of Alternative Isoforms in the Human Proteome
Source: Cell Rep. Author manuscript; Available in PMC 2020 Jan 15. (PMC6961840; doi:10.1016/j.celrep.2019.11.026)
Supplement: 2 [file NIHMS1546469-supplement-2.zip › DF1/PXD010154/Testis/Testis_4_RHOT2_LSDQDLDQALSDEELNAFQQK.pdf]

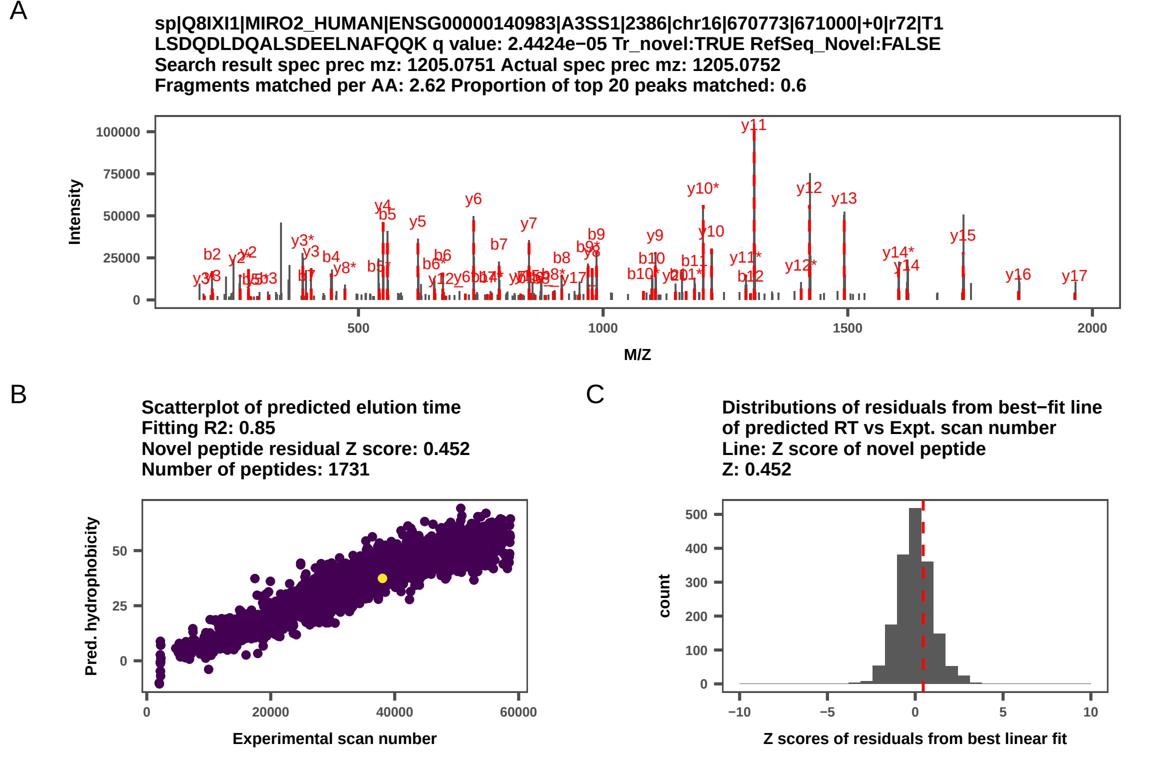
A
sp|Q8IXI1|MIRO2_HUMAN|ENSG00000140983|A3SS1|2386|chr16|670773|671000|+0|r72|T1
LSDQDLDQALSDEELNAFQQK q value: 2.4424e−05 Tr_novel:TRUE RefSeq_Novel:FALSE
Search result spec prec mz: 1205.0751 Actual spec prec mz: 1205.0752
Fragments matched per AA: 2.62 Proportion of top 20 peaks matched: 0.6
Intensity
100000
75000
50000
25000
0
M/Z
500
1000
1500
2000
y11
y10*
y12
y13
y6
y4
b5
y5
y7
b9
y10
y9
y15
y3*
y3
b2
y2*
y2
b4
b7
y8*
b5*
b6
b6*
b8
b10
b11
y11*
y12*
y14*
y14
y16
y17
b12
B
Scatterplot of predicted elution time
Fitting R2: 0.85
Novel peptide residual Z score: 0.452
Number of peptides: 1731
Pred. hydrophobicity
50
25
0
Experimental scan number
0
20000
40000
60000
C
Distributions of residuals from best−fit line
of predicted RT vs Expt. scan number
Line: Z score of novel peptide
Z: 0.452
count
500
400
300
200
100
0
Z scores of residuals from best linear fit
−10
−5
0
5
10
